# Prediction Model for COVID-19 Vaccination Intention among the Mobile Population in China: Validation and Stability

**DOI:** 10.3390/vaccines9111221

**Published:** 2021-10-21

**Authors:** Fan Hu, Ruijie Gong, Yexin Chen, Jinxin Zhang, Tian Hu, Yaqi Chen, Kechun Zhang, Meili Shang, Yong Cai

**Affiliations:** 1 School of Public Health, Shanghai Jiao Tong University School of Medicine, Shanghai 200025, China; hufan@sjtu.edu.cn (F.H.); cynthia-dt@sjtu.edu.cn (R.G.); chenyexin888@sjtu.edu.cn (Y.C.); jjjxxzhang@sjtu.edu.cn (J.Z.); 2 Xuhui Center for Disease Control and Prevention, Shanghai 200237, China; 3 Longhua Center for Disease Control and Prevention, Shenzhen 518100, China; ht1137571641@126.com (T.H.); chloe4697@163.com (Y.C.); 4 Sanlin Community Health Service Center, Shanghai 200124, China

**Keywords:** COVID-19, vaccination intention, nomogram, prediction model, model validation

## Abstract

Since China’s launch of the COVID-19 vaccination, the situation of the public, especially the mobile population, has not been optimistic. We investigated 782 factory workers for whether they would get a COVID-19 vaccine within the next 6 months. The participants were divided into a training set and a testing set for external validation conformed to a ratio of 3:1 with R software. The variables were screened by the Lead Absolute Shrinkage and Selection Operator (LASSO) regression analysis. Then, the prediction model, including important variables, used a multivariate logistic regression analysis and presented as a nomogram. The Receiver Operating Characteristic (ROC) curve, Kolmogorov–Smirnov (K-S) test, Lift test and Population Stability Index (PSI) were performed to test the validity and stability of the model and summarize the validation results. Only 45.54% of the participants had vaccination intentions, while 339 (43.35%) were unsure. Four of the 16 screened variables—self-efficacy, risk perception, perceived support and capability—were included in the prediction model. The results indicated that the model has a high predictive power and is highly stable. The government should be in the leading position, and the whole society should be mobilized and also make full use of peer education during vaccination initiatives.

## 1. Introduction

As of 11 April 2021, according to the World Health Organization, there are more than 135 million cases of coronavirus disease 2019 (COVID-19) infections and more than 2.91 million deaths worldwide [1]. To establish an immune barrier and control the COVID-19 epidemic in the population, COVID-19 vaccination is a key public health strategy. During the previous phase of the epidemic, countries focused on the development of a COVID-19 vaccine, and after the successful launch of the vaccine, vaccination rates have become a global concern as the key to population immunization effectiveness. Acceptability of the COVID-19 vaccine may vary according to several demographic characteristics, among which the individual willingness to vaccinate plays an important role, and a high individual willingness to vaccinate is more likely to increase the acceptability of the COVID-19 vaccine. Mobile populations, as an important group in the spread of the epidemic, should receive extra attention in terms of their vaccination intentions and vaccination rates.

At the end of 2020, China launched the COVID-19 vaccination, and the government has been encouraging the general public to get vaccinated for free. By 18 April 2021, China’s total vaccination amount of COVID-19 vaccines was 155 million doses [2], ranking second in the world, while the United States was the first. However, the coverage rate of the COVID-19 vaccine is only 10%, ranking 49th in the world, which is far lower than 77.6% of the coverage rate needed to build an effective population immunization. Previous studies have shown that personal psychology, social support and behavior change factors play an important role in the intention rate of COVID-19 vaccination. Guidry et al. found that insurance, education, susceptibility to COVID-19, vaccine benefits, scores of vaccine barriers, subjective norms scores, attitudes towards the vaccine and self-efficacy were significant predictors of COVID-19 vaccination intentions [3]. Meanwhile, concerns about the hasty development of the COVID-19 vaccine [3], fear of needles, concerns about pain during vaccination, lack of family support and concerns about side effects were identified as the reasons for vaccination hesitation [4]. A cross-sectional study in China found that a high perception of the benefits and low perceived barriers to vaccination were the two most important factors influencing a clear intention to vaccinate with COVID-19 [5]. In addition, vaccination history, doctor’s advice and convenience of the vaccination all influenced respondents’ intention to receive the vaccine immediately, and safety information about vaccination published by an authoritative institution could effectively increase vaccination intentions [6]. Another survey of 27 cities in China showed that income and occupation were important determinants of willingness to receive the free COVID-19 vaccine, and the occupational population with a high risk of COVID-19 infection was the most willing to receive the vaccine [7]. By the end of April, the highest vaccination rate of the COVID-19 vaccine in China was for medical personnel, whose vaccination rate exceeded 80%, but the situation of vaccination for the mobile population was not optimistic. Improving the vaccination rate of the mobile population is the key to prevent virus transmission, and the first thing is to improve the vaccine willingness of the mobile population.

There are many studies at home and abroad that have identified factors influencing vaccination willingness through a logistic regression analysis [7,8] and Pearson’s chi-square test in the general population. Graffigna et al. [9] developed two pathway models in their study of COVID-19 vaccination intentions. By examining the effect of health participation on the willingness to vaccinate against COVID-19, a direct pathway between health participation and willingness to vaccinate was established, as well as another indirect pathway mediated by the general attitudes towards vaccination. The study suggests that a multifactorial health behavior model should be used as a basis for research and interventions concerning vaccination intentions [9]. However, there are few surveys on the vaccination intention of the mobile population. In this study, the key factors influencing the vaccination intention of the COVID-19 vaccine were investigated by establishing a clinical prediction model, and the government and related departments can take measures to improve the vaccination intention of the mobile population based on these key factors, so as to improve the vaccination rate of the whole people.

## 2. Materials and Methods

### 2.1. Subjects

This cross-sectional survey was conducted in April 2021 in Longhua District, Shenzhen, China, and ethical approval was obtained from the Ethics Committee of School of Public Health, Sun Yat-sen University.

A stratified multi-stage sampling method was used to recruit the study population. The research team randomly selected eight factories out of 1805 and two to three workshops from each factory, resulting in 859 full-time employees aged ≥18 years who were all mobile to participate in the study. The study guaranteed anonymity, and study participants had the right to withdraw at any time and without consequences. Written informed consent was obtained from all participants before the study was conducted. Finally, 782 valid questionnaires were collected. 

### 2.2. Measurement

The questionnaire design for this study was based on the theoretical framework of the Reasoned Action Approach [10] and was informed by a questionnaire authorized by Visser [11].

The main outcome of the questionnaire was the intention to receive the COVID-19 vaccine within 6 months. Participants that answered “yes” were classified as having vaccination intention, and those who answered either “no” or “unsure” were classified as not having vaccination intention. Their personal, psychosocial and behavioral determinants were measured (Table 1). The personal determinants included demographic characteristics such as gender, age, income and education. For the psychosocial determinants survey component, anxiety was measured using the Chinese version of Generalized Anxiety Disorder (GAD-7) (Cronbach’s α = 0.960) [12]. Depression was measured by the Chinese version of the Patient Health Questionnaire (PHQ-9) (Cronbach’s α = 0.948) [13]. Interpersonal needs were measured by the Chinese version of the Interpersonal Needs Questionnaire (INQ-15) (Cronbach’s α = 0.829) [14].

All items in the behavioral determinants survey section were rated from 1 “strongly disagree” to 7 “strongly agree” (Cronbach’s α = 0.935). General beliefs about vaccination (i.e., I take vaccinations for granted) and past experiences (i.e., I remember which vaccines I have received) constitute the general perceptions about vaccination; self-efficacy (i.e., I would get myself vaccinated with the COVID-19 vaccine if offered), risk perception (i.e., I think there is a risk of serious side effects, such as severe allergic reactions, if I get the COVID-19 vaccine), fear of vaccination (i.e., I am afraid of the injection), perceived benefits (i.e., if I get the COVID-19 vaccine, I am doing so primarily to protect myself), and perceived barriers (i.e., I think the COVID-19 vaccine has a limited duration of protection) comprise the personal perceptions toward COVID-19 vaccination. The perceived support consisted of nine items (i.e., my family believes I should get the COVID-19 vaccine). The ability to receive the COVID-19 vaccine consisted of eight items (i.e., I can tolerate my fear of needles to receive the COVID-19 vaccine). See Table 1.

### 2.3. Statistical Analyses

The statistical analysis of the data in this study was conducted through R software (version4.1.0, https://www.r-project.org, accessed on 24 May 2021). First, the participants were divided into a training set and a testing set for external validation conformed to the ratio of 3:1 with R software. A descriptive statistical analysis, *t*-test, and chi-square test were performed. The Least Absolute Shrinkage and Selection Operator (LASSO) regression analysis was used to screen the 16 independent variables of the training set to identify the factors. Area under curve (AUC), as one of the measures of LASSO, was used to specify the target covariates that were minimized when cross-validating the selected model. 1-standard error (1-SE) was used to obtain a model with excellent performance and a minimum number of independent variables. After features with nonzero coefficients in the LASSO regression analysis were selected, a multivariate logistic regression analysis was performed on the training set to build the prediction model, which was presented as a nomogram. Based on the training set and the testing set, the Receiver Operating Characteristic (ROC) curve, Kolmogorov–Smirnov (K-S) test, Lift test and Population Stability Index (PSI) were performed to test the validity and stability of the model and summarize the validation results.

## 3. Results

### 3.1. Demographic Characteristics of Participants

Finally, 782 valid data were included in the study, including 363 males and 419 females. The average age of the participants was 32.00 (28.00–38.00) years old. Three hundred and fifty-six (45.52%) of the participants had the intention of receiving the vaccination, 87 (11.13%) were refused to get vaccinated and 339 (43.35%) were unsure. Finally, 426 were classified as having no intention of receiving the vaccination. For external validation, the total data was divided into a training set (588 participants) and a testing set (194 participants) at a ratio of approximately 3:1. The training set contained 273 males and 351 females with an average age of 32.00 (28.00–38.00) years old. There were 272 (46.26%) participants willing to get vaccinated. The testing test included 90 males and 104 females, with an average age of 33.00 (28.00–39.00) years old, and 84 (43.30%) participants had the intention to get vaccinated. The specific demographic indicators and clinical characteristics of the groups are presented in Table 2.

### 3.2. Predictive Model Construction

**Figure 1:**
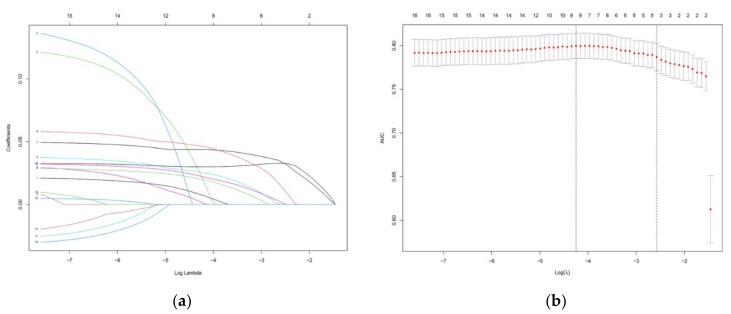
Feature selection using the LASSO regression model for COVID-19 vaccination intentions. (**a**) Variable selection by the LASSO binary logistic regression model. A coefficient profile plot was constructed against the log (lambda) sequence. Four variables with nonzero coefficients were selected by deriving the optimal lambda. (**b**) Following verification of the optimal parameter (lambda) in the LASSO model, we plotted the partial likelihood deviance (AUC) curve versus the log (lambda) and drew dotted vertical lines based on the 1 standard error criteria: the LASSO Least Absolute Shrinkage and Selection Operator.

**Figure 2:**
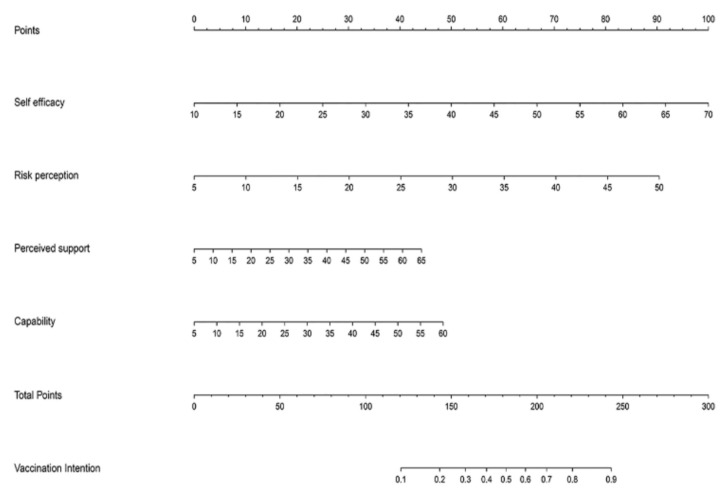
Developed nomogram for the COVID-19 vaccination intentions. Important features of self-efficacy, risk perception, perceived support and capability for the nomogram prediction model.

The features with nonzero coefficients selected by LASSO include self-efficacy, risk perception, perceived support and capability. The lambda.1se of these indicators are 0.034, 0.014, 0.002 and 0.033, respectively (Figure 1a,b). The significant factors selected were included in the logistic regression analysis. The coefficients of all the included variables are shown in Table 3. Then, the nomogram diagrams corresponding to the prediction model were established (Figure 2).

### 3.3. Prediction Model Validation

**Figure 3:**
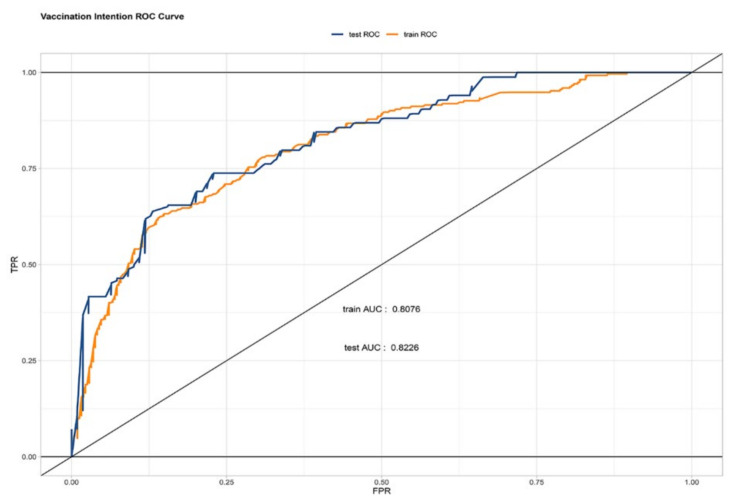
The pooled AUC of the ROC curve in the training set and test set. Receiver operating characteristic curve (ROC) validation of the vaccination intention nomogram prediction. The *y*-axis represents the true positive rate of the prediction, and the *x*-axis represents the false positive rate of the prediction. The orange line represents the performance of the model in the training set, and the blue line represents the performance of the model in the test set.

**Figure 4:**
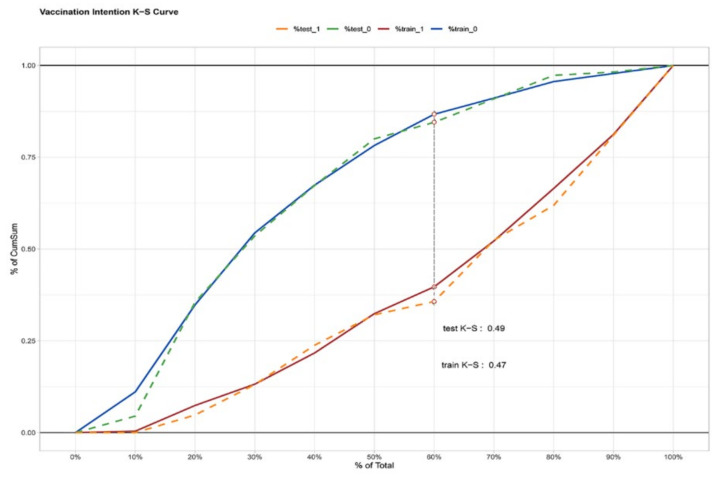
The K–S values in the training set and test set. The K–S values of the vaccination intention prediction model. The red line represents the positive performance of the model in the training set, and the blue line represents the negative performance of the model in the training set. The green line represents the negative performance of the model in the test set, and the orange line represents the positive performance of the model in the test set.

**Figure 5:**
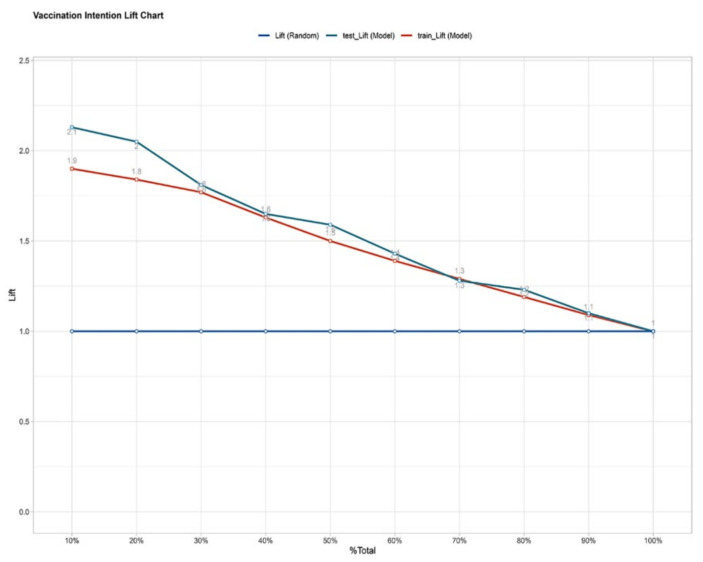
The lift chart in the training set and test set. The lift chart of the vaccination intention prediction model. The bule line is random, the red line represents the performance of the model in the training set, and the green line represents the performance of the model in the test set.

**Figure 6:**
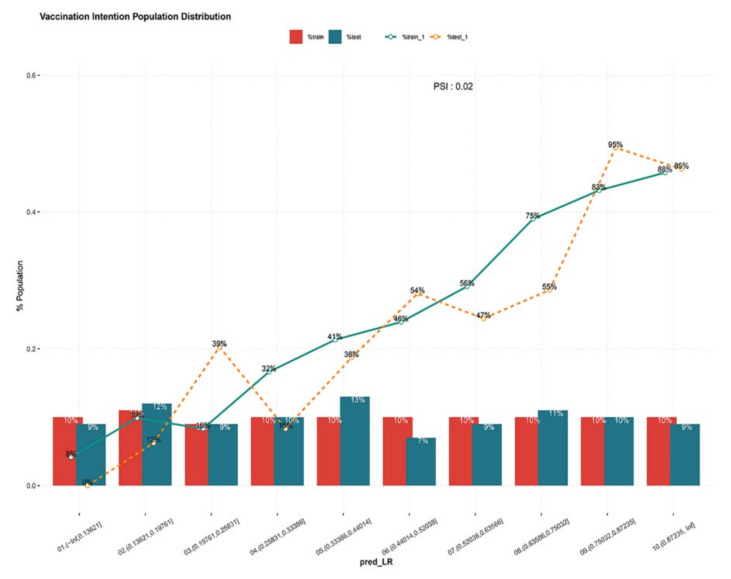
The PSI in the training set and test set. The PSI of the vaccination intention prediction model. The green line represents the performance of the model in the test set, and the orange line represents the performance of the model in the training set.

The ROC of the prediction model was reported in Figure 3. The AUC value of the model was 0.808 in the training set and 0.823 in the testing set. The AUC values based on both sets of data were greater than 0.8, indicating that the prediction model had a good algorithm. The K–S values were 0.47 and 0.49 in the training set and testing set, respectively (Figure 4). The validation results were all greater than 0.2, indicating that the risk differentiation ability of the model established in this study was strong. From the results of the Lift test shown in Figure 5, it can be found that the values of the training set and testing set were above 1, with a large increase in the index, which proved that the model had a high predictive power. The PSI value of 0.02 in this study was less than 0.1, indicating that the model was highly stable (Figure 6). Finally, all the results are aggregated and presented in Table 4. To summarize the results from the above verification, this model has a good prediction ability.

## 4. Discussion

In our study, the reported vaccination intention within 6 months was 45.52%, only half the results of previous studies [5,6,15]. This result indicated that there exists so-called vaccination hesitancy during the campaign of COVID-19 vaccination. According to the prediction model established in our study, self-efficacy, risk perception, perceived support and capability were the predictors of vaccination intention.

The Health Belief Model, the Theory of Planned Behavior and discrete choice experiments were commonly used in previous studies [3,5,16]. The Reasoned Action Approach was firstly used in understanding COVID-19 vaccination intentions. Meanwhile, our study used novel statistical methods to predict the vaccination intentions within the next 6 months in China. The variables were screened using the LASSO regression analysis. The ROC, K–S test, Lift test and PSI were performed to verify the accuracy and stability of the prediction model.

### 4.1. Association of Mobile Population and Vaccination Intention

The results of this study are similar to a previous study reported in Qatar, with a rate of 60% [17]. Since the mobile population faces more barriers to vaccination—for example, restricted access to healthcare, social exclusion and vaccination initiatives—they are commonly regarded with lower routine vaccine uptake rate and a higher risk of dissemination and infection [18]. Thus, urgent action should be taken for inclusion of the mobile population in the vaccination campaign.

### 4.2. Association of Self-Efficacy and Vaccination Intention

During the study, the pandemic was largely controlled in China. It seemed that there was no necessity to get the COVID-19 vaccine, which meant that the public believed that they would were not enough perceived benefits from vaccination. Since the results from previous studies showed that the higher the efficacy, the higher the vaccination intention, with a vaccine efficacy of 90% or above, 84.3% would get a vaccination within 6 months [6]. The efficacy results of Sinovac, Sinopharm (Beijing, Wuhan) were 50.4%, 79.3% and 72.5% efficacy rates in the phase 3 trials, lower than the mRNA vaccines at efficacy rates from 94 to 95% [19]. Considering more types of COVID-19 vaccines will be available on the market in the near future, those who prefer vaccines with a higher efficacy rate would be hesitated toward the current vaccines.

### 4.3. Association of Risk Perception and Vaccination Intention

One of the most common concerns about vaccinations is whether there are adverse effects. The typical examples are a reluctance to accept the measles vaccine in parts of Europe, wrongly reported as causing autism [20], and the human papillomavirus vaccine in Japan after unfounded sensational reports of so-called “adverse events” were published by the Japanese media [21]. Regarding the COVID-19 vaccines, the mRNA vaccine is so novel that even the safety profile showed no safety concerns [22], but the public still worries about its long-term adverse effects. Additionally, the vaccine developed by Oxford, in conjunction with AstraZeneca, has been paused several times worldwide [23]. Recently, it was reported to associate with thrombus. Fortunately, the vaccines now available in China are an inactivated, nonreplicating viral vector and recombinant subunit [24]. Up to now, no serious adverse effects of those vaccines have been reported. 

### 4.4. Association of Perceived Support and Vaccination Intention

While most researchers focus on the influence of antivaccine reports or groups, few researchers have studied the association of perceived support and vaccination hesitancy [25]. Previous studies have emphasized the role of healthcare providers and social media in eliminating vaccine hesitancy [23,26], while the impact of family, friends and significant others is often ignored. This is easy to understand, since once a person gets the COVID-19 vaccination, he/she will be protected from infection but, also, so will those close be safe from being infected by him/her.

### 4.5. Association of Capability and Vaccination Intention

Previous studies have shown that capability is an important predictor of vaccination intention [11,27]. This agrees with our results on the COVID-19 vaccination. It could be easily understood that the definition of vaccination intention in our study is to get a COVID-19 vaccine within 6 months, which includes not only the willingness to get the vaccine but, also, the capability to get the COVID-19 vaccine. Thus, although capability was not a significant factor in the results of the model, it was still included in the prediction model for intention. 

### 4.6. Limitations

First, only one cross-sectional survey has been conducted at present. A follow-up study is needed to investigate the behavior of whether the participant is vaccinated. Second, if the population sample can be expanded to the entire population, the representativeness will be better. Thus, more data will be collected in future research.

## 5. Conclusions

Self-efficacy, risk perception, perceived support and capability are meaningful predictors for COVID-19 vaccination. Thus, health education on COVID-19 vaccination is necessary. The benefits and possible adverse effects of the vaccination should be fully informed. The government should be in the leading position, and the whole society should be mobilized and, also, making full use of peer education during vaccination initiatives.

## Figures and Tables

**Table 1:** Personal, psychosocial and behavioral determinants of COVID-19 vaccination intention.

Determinants	Determinants Measured	Number of Items
Personal determinants	Demographic characteristics	4
Psychosocial determinants		
	Depression	9
	Anxiety	7
	Interpersonal need	15
Behavioral determinants		
General view on vaccination	General beliefs about vaccination	4
	Past experience	3
Views on COVID-19 vaccination	Self-efficacy	10
	Risk perception	6
	Fear of vaccination	5
	Perceived benefits	4
	Perceived barriers	2
Perceived support	Perceived support	9
Capability	Capability	8
Intention	Intention	1

Note: Structured questionnaire descriptions, including variable names and quantities.

**Table 2:** Characteristics of the participants in the different groups.

Items	Total (*n* = 782)	Training Set	Testing Set	*p*-Value
		(*n* = 588)	(*n* = 194)	
Age (years old)	32 (28,38)	32 (28,38)	33 (28,39)	0.681
Sex, *n* (%)				0.993
Male	363 (46.4)	273 (46.4)	90 (46.4)	
Female	419 (53.6)	315 (53.6)	104 (53.6)	
Education, *n* (%)				0.533
Junior middle school and below	48 (6.1)	34 (5.8)	14 (7.2)	
High school	224 (28.6)	175 (29.8)	49 (25.3)	
Junior college	209 (26.7)	161 (27.4)	48 (24.7)	
Undergraduate	238 (30.4)	172 (29.3)	66 (34.0)	
Postgraduate and above	63 (8.1)	46 (7.8)	17 (8.8)	
Income				0.999
Extremely high	8 (1.0)	6 (1.0)	2 (1.0)	
High	48 (6.1)	36 (6.1)	12 (6.2)	
Middle	575 (73.5)	431 (73.3)	144 (74.2)	
Low	122 (15.6)	93 (15.8)	29 (14.9)	
Extremely low	29 (3.7)	22 (3.75)	7 (3.6)	
Anxiety	9 (7,14)	9 (7,14)	9 (7,14)	0.748
Depression	12 (9,18)	12 (9,18)	13 (9,18)	0.538
Interpersonal need	37 (25,51)	37 (25,51)	35 (25,50)	0.263
General beliefs	22 (18,26)	22 (18,26)	22 (18,26)	0.796
Past experience	14 (11,15)	14 (11,15)	14 (12,15)	0.231
Self-efficacy	51 (41,60)	51 (42,60)	51 (40,60)	0.941
Risk perception	30 (26,35)	30 (26,35)	30 (26,35)	0.694
Fear of vaccination	18 (15,24)	18 (15,24)	19 (15,24)	0.605
Perceived benefits	25 (21,28)	25 (21,28)	24.5 (21,28)	0.696
Perceived barriers	7 (5,9)	7 (6,9)	7 (5,9)	0.319
Perceived support	38 (34,49)	38 (35,50)	37 (32,47)	0.274
Capability	40.5 (32,50)	40 (32,50)	42 (33,50)	0.492

Note: All variable values in table have the median in parenthesis, unless indicated otherwise.

**Table 3:** Logistic regression analysis of the predictors for vaccination intention.

Determinants	β-Coefficient	*p*-Value	OR (95% CI)
Self-efficacy	0.060	<0.001 ^a^	1.062 (1.030–1.095)
Risk perception	0.072	<0.001 ^a^	1.075 (1.046–1.105)
Perceived support	0.026	0.033 ^a^	1.027 (1.002–1.052)
Capability	0.032	0.073	1.032 (0.997–1.068)

Note: Data presented as the β-coefficient and OR (95%CI). Statistical significance recognized as *p* < 0.050 and denoted by ^a^.

**Table 4:** Summary of the model validation results.

PSI	Test_Lift	Train_Lift	Test_K-S	Train_K-S	%Test_cumB	%Test_cumG	%Train_cumB	%Train_cumG	%Test_B	%Train_B	%Test	%Train	#Test	#Train	#Total
0	1	1	0.16	0.15	0	0.16	0.02	0.17	0	0.08	0.09	0.1	18	59	77
0	1.1	1.09	0.32	0.28	0.04	0.35	0.07	0.34	0.12	0.19	0.12	0.11	24	67	91
0	1.23	1.19	0.34	0.38	0.12	0.45	0.1	0.48	0.39	0.16	0.09	0.09	18	51	69
0	1.28	1.29	0.45	0.44	0.15	0.6	0.17	0.6	0.16	0.32	0.1	0.1	19	59	78
0.01	1.43	1.39	0.48	0.46	0.26	0.75	0.25	0.72	0.36	0.41	0.13	0.1	25	59	84
0.01	1.59	1.5	0.45	0.46	0.35	0.8	0.35	0.82	0.54	0.46	0.07	0.1	0.13	59	72
0	1.65	1.63	0.44	0.42	0.44	0.88	0.47	0.9	0.47	0.56	0.09	0.1	17	59	76
0	1.81	1.33	0.39	0.31	0.58	0.97	0.64	0.95	0.55	0.75	0.11	0.1	22	59	81
0	2.05	1.84	0.17	0.16	0.81	0.98	0.82	0.98	0.95	0.83	0.1	0.1	20	59	79
0	2.13	1.9	0	0	1	1	1	1	0.89	0.88	0.09	0.1	18	57	75

## Data Availability

The data presented in this study are available upon reasonable request from the corresponding author.
